# Post-exercise hypotension and heart rate variability response after water- and land-ergometry exercise in hypertensive patients

**DOI:** 10.1371/journal.pone.0180216

**Published:** 2017-06-28

**Authors:** Danilo Sales Bocalini, Marco Bergamin, Alexandre Lopes Evangelista, Roberta Luksevicius Rica, Francisco Luciano Pontes, Aylton Figueira, Andrey Jorge Serra, Emilly Martinelli Rossi, Paulo José Ferreira Tucci, Leonardo dos Santos

**Affiliations:** 1 Translational Physiology Laboratory, Post-Graduation Program in Physical Education, São Judas Tadeu University, São Paulo, Brazil; 2 Post-Graduation Program in Aging, São Judas Tadeu University, São Paulo, Brazil; 3 Sport and Exercise Medicine Division, Department of Medicine, University of Padova, Padova, Italy; 4 Department of Physical Education of University of São Paulo, São Paulo, Brazil; 5 School of Arts, Sciences and Humanities of University of São Paulo, São Paulo, Brazil; 6 Post-Graduation in Biophotonic applied in Healthy Science of Nove de Julho University, São Paulo, Brazil; 7 Department of Physiological Sciences, Federal University of Espirito Santo, Espirito Santo, Brazil; 8 Cardiology division of Department of Medicine, Federal University of São Paulo - São Paulo Medical School, São Paulo, Brazil; University of Debrecen, HUNGARY

## Abstract

**Background:**

systemic arterial hypertension is the most prevalent cardiovascular disease; physical activity for hypertensive patients is related to several beneficial cardiovascular adaptations. This paper evaluated the effect of water- and land-ergometry exercise sessions on post-exercise hypotension (PEH) of healthy normotensive subjects versus treated or untreated hypertensive patients.

**Methods:**

Forty-five older women composed three experimental groups: normotensive (N, n = 10), treated hypertensive (TH, n = 15) and untreated hypertensive (UH, n = 20). The physical exercise acute session protocol was performed at 75% of maximum oxygen consumption (VO_2max_) for 45 minutes; systolic (SBP), diastolic (DBP) and mean (MBP) blood pressure were evaluated at rest, peak and at 15, 30, 45, 60, 75 and 90 minutes after exercise cessation. Additionally, the heart rate variability (HRV) was analyzed by R-R intervals in the frequency domain for the assessment of cardiac autonomic function.

**Results:**

In both exercise modalities, equivalent increases in SBP were observed from rest to peak exercise for all groups, and during recovery, significant PEH was noted. At 90 minutes after the exercise session, the prevalence of hypotension was significantly higher in water- than in the land-based protocol. Moreover, more pronounced reductions in SBP and DBP were observed in the UH patients compared to TH and N subjects. Finally, exercise in the water was more effective in restoring HRV during recovery, with greater effects in the untreated hypertensive group.

**Conclusion:**

Our data demonstrated that water-ergometry exercise was able to induce expressive PEH and improve cardiac autonomic modulation in older normotensive, hypertensive treated or hypertensive untreated subjects when compared to conventional land-ergometry.

## Introduction

Systemic arterial hypertension is the most prevalent cardiovascular disease among adults [1], representing numerous deaths per year [2,3]. Similar data have been found in the Brazilian population, showing a 24.4% prevalence of hypertension that increased to 63.2% in the population aged over 65 years old [4,5].

It is well known that pharmacological treatment associated with behavioral-educational approaches both reduce cardiovascular and cerebrovascular morbidity-mortality. However, the major strategy to prevent the impact of hypertension is related to educational lifestyle changes, such as better nutrition, smoking cessation, reduced alcohol consumption and regular physical activity or exercise. Behavioral-educational intervention is a non-pharmacological strategy to control hypertension, and physical activity practice may lead to other behavior modifications (e.g., smoking, alcohol intake, and high fat and/or sugar diets) that impact the morbidity-mortality rate, especially if aerobic exercise [6] is associated with pharmacological treatment [7].

Several cardiovascular adaptations are related to regular physical activity, such as post-exercise hypotension (PEH). Clinically, PEH contributes to blood pressure control as a result of either chronic or acute cardiovascular adaptations in hypertensive and normotensive subjects [8]. Some mechanisms have been described, such as decreased peripheral resistance and sympathetic activity, as well as changes in stroke volume, beta-adrenergic receptors and endothelial modulation [7,9]. Interestingly, different PEH responses are found in each exercise modality such as land walking, cycling, treadmill walking or strength training [9–17] as a result of differences in their metabolic and cardiovascular demand.

Aerobic exercise practice in water is highly recommended for the elderly [18–21] because of the low load and mechanical stress on weight-bearing joints and muscles; water exercises also seem to have the added benefit of a reduction in pain [19]. This type of activity is indicated in rehabilitation and therapeutic procedures and is included in exercise programs for cardiovascular and muscular fitness improvement, particularly for adults and elderly with movement limitations [20,21].

Physiological adaptations are reported in both water- and land-based exercises [21–27], although the magnitudes of the effects are different, mainly as a result of the greater decrease in the sympathetic drive, catecholamine release and peripheral vascular resistance in water-based exercise. Higher suppression in vasopressin and renin-angiotensin systems is also reported in water-based exercise when compared to physical activities on land. Notwithstanding, few studies investigated PEH effectiveness after water-based exercise [15,16]. Recently, Rodriguez et al [15] described significant PEH in normotensive women after exercise in the water. In hypertensive subjects, Pontes Junior et al [16] found similar PEH performance when comparing land- with water-based exercise. Considering the scarce and inconclusive data that are published in the literature, it is still relevant to identify what would be the actual effects of water- or land-based aerobic exercise on normotensive and hypertensive adult subjects. Thus, in this study we aimed to compare the effectiveness of water-based cycle ergometry with land-based cycle ergometry on the post-exercise hypotension response in normotensive, treated and untreated hypertensive subjects.

## Materials and methods

### Subjects

Ten normotensive female subjects (N) and thirty-five hypertensive female patients were included after criteria selection. Hypertensive patients were divided into treated (TH, n = 15) or untreated (UH, n = 20) groups according to whether the patients received optimized therapy or not, respectively. All included women had no physical limitations. Written informed consent was obtained from all participants; the procedures were performed according to the principles of the Declaration of Helsinki (www.wma.net/e/policy/b3.htm) and were approved by the Human Research Ethics Committee of Federal University of São Paulo—Paulista Medicine School (834.135/2014). Subjects with recent hospitalization, symptomatic cardiorespiratory disease or cardiac alterations, metabolic syndrome, severe renal or hepatic disease, cognitive impairment, debilitating condition, obesity with inability to exercise and any other medical contraindications to physical exercise were excluded.

### Body composition

The anthropometric variables were assessed by an experienced researcher following the Serra [28] protocol. Stature was measured (in m) to the nearest 0.1 cm criteria by a Cardiomed stadiometer (264 model, Parana, Brazil). Body mass was determined (in kg) to the nearest 0.1 kg using a Filizola scale (Personal Line 150 model), and body mass index (BMI) was calculated (kg/m^2^). Body composition was determined through skinfold thickness using the Lange Skinfold Caliper (Lafayette Instruments, USA). Fat mass (FM) was determined by the following equation (FM = %Fat x weight ÷ 100) and the lean mass was estimated by the proportion of fat mass from the total body mass.

### Blood pressure and heart rate

Blood pressure (BP) was measured between 8–10 am, and subjects remained seated for 20 minutes before the protocol [29]. Systolic (SBP) and diastolic BP (DBP) and heart rate (HR) were measured before, during, and immediately after each exercise session using an automated non-invasive BP monitor (Microlife 3AC1-1PC, Microlife, Widnau, Switzerland). Additionally, the mean arterial blood pressure (MBP) was calculated using the equation MBP = DBP + [SBP–DBP]/3 and the rate-pressure product (RPP) was evaluated according to the following equation: RPP = HR * SBP. Resting heart rate and SBP and DBP were assessed on land and in water immersion conditions. In the water, the subjects remained standing for 15 minutes to serve as the control session. After exercise sessions, blood pressure and heart rate were evaluated at 15, 30, 45, 60, 75 and 90 minutes with subjects seated outside the pool. The subjects were instructed to not perform any physical activity and not consume caffeine or alcohol 24 hours before the evaluation.

### Heart rate variability

The heart rate variability (HRV) was evaluated according to previous publications [30]. Briefly, the five minute beat-to-beat HR was recorded using a frequency monitor (Polar Electro Oy S810i, Kempele, Finland) at a sample rate of 1,000 Hz before (at rest) and ninety minutes after the exercise session, with all measurements captured on land to avoid water immersion-related changes. The RR intervals were recorded and transferred to a computer using software (Polar Precision Performance, version 3.02.007, Kempele, Finland). Data were edited manually by visual inspection in an attempt to avoid artifacts and to avoid affecting the signal. Spectral analysis was performed to assess the auto regressive RR interval variability using the HRV analysis software (version 1.1, Finland). The components of the HRV were obtained by integration of successive heart rate ranges distributed as follows: very low frequency component 0.003–0.04 Hz (VLF, representing the influence of the peripheral vasomotor and renin-angiotensin systems); low frequency component 0.04–0.15 Hz (LF, corresponding to sympathetic and parasympathetic); and high-frequency component 0.15–0.4 Hz (HF: corresponding to vagal input). The results are presented in absolute and normalized powers of the bands excluding VLF, and the LF/HF ratio was calculated to characterize the sympathovagal balance.

### Acute exercise session

Protocols for ergometer biking exercise (on land- or in water-based exercise) were designed to compare the PEH after each modality. Subjects were evaluated with a treadmill exhaustion incremental performance test according to Balke’s protocol and a previous publication from our group [19,20]. The maximum oxygen uptake (VO_2max_) was recorded every 30 seconds using a Quinton QMC 000350 (Bothell, WA) gas analyzer. The treadmill test was interrupted when the subject reached their maximum heart rate (HR_max_) minus 10 beats per minute of HR predicted-age criteria. Heart rate was monitored every 30 seconds during the test by 12-lead electrocardiography (Quinton, Q710 Bothell, WA). The exercise prescription was based on VO_2max_ evaluation to 75% intensity as described by Forjaz [8], with a 45-minute session duration. The subjects were randomly distributed into the two protocols (land- and water-based exercise) in the two different sessions (48 hours between sessions).

The protocol for water-based exercise conditions was performed in a pool with the temperature adjusted to 30±1°C, as recommended by the Aquatic Exercise Association [31] and the depth of the water was set at the xiphoid process level. The resistance characteristic for the water-based exercise was a result of the natural property of water that was increased following exercise prescription by pedaling velocity. The exercise intensity was monitored by HR (Accurex Plus, Polar Electro, Kempele, Finland) as well as perceived exertion when it reached 6–20 points on the Borg Scale.

### Statistical analyses

Statistical analyses were conducted by SPSS for Windows software (version 15.0, SPSS Inc., Chicago, Illinois, USA). For descriptive statistics, data are presented as mean ± SD, and for inferential statistics, date are presented as mean ± SEM. The D'Agostino-Pearson test was applied for Gaussian distribution analysis. The comparisons between groups were assessed using one-way and two-way ANOVA with repeated measures and Bonferroni’s post hoc tests. The confidence value was established at p< 0.05.

## Results

The three groups did not differ in age or anthropometric parameters; the hemodynamic parameters as measured on land are presented in Table 1, evidencing increased SBP, DBP and heart rate pressure product (HPP) in the UH group when compared to the N group. ACE-inhibitors were the most prevalent in the TH group; the TH group exhibited no differences in the BP levels in relation to the N group.

**Table 1:** General profile of the subjects.

Parameters	Normotensive	Treated hypertensive	Untreated hypertensive
Age (years)	62 ± 2	64 ± 2	66 ± 2
*Anthropometry*			
Body weight (kg)	71 ± 4	72 ± 3	74 ± 2
Height (m)	1.60 ± 1	1.62 ± 1	1.60 ± 1
BMI (kg/m^2^)	28 ± 2	28 ± 1	29 ± 1
Body fat (%)	32 ± 2	33 ± 1	34 ± 1
Fat mass (kg)	39 ± 4	39 ± 3	40 ± 2
Lean mass (kg)	32 ± 2	33 ± 1	34 ± 1
*Hemodynamics*			
HR (bpm)	77 ± 4	77 ± 5	84 ± 4
SBP (mmHg)	121 ± 3	122 ± 2	155 ± 4*
DBP (mmHg)	80 ± 4	82 ± 4	92 ± 3*
MBP (mmHg)	94 ± 2	95 ± 2	110 ± 3
HPP (bpm*mmHg)	9427 ± 385	9598 ± 375	130298 ± 6887*
*Medicine*			
Period of medicine (years)	---	8 ± 4	---
Diuretics (%)	---	56%	---
ACE-inhibitors (%)	---	88%	---
Calcium blocker (%)	---	50%	---

Values are presented as mean *±* SD of normotensive, treated hypertensive and untreated hypertensive subjects for body mass index (BMI), heart rate (HR), systolic blood pressure (SBP), diastolic blood pressure (DBP), mean blood pressure and heart rate pressure product (HPP). One-way ANOVA was used, followed by Bonferroni’s post hoc test.

*p<0.001 indicates statistically significant differences between normotensive and treated hypertensive subjects on land.

When evaluating the potential effects of water immersion on the baseline hemodynamics (Table 2), no differences were found in BP or HR in land versus water immersion conditions at rest, suggesting that 15 min of immersion was not enough to significantly alter hemodynamic parameters. Thus, SBP and HPP from the UH group were higher than in the N and TH subjects on land as well as in water conditions.

**Table 2:** Hemodynamic parameters at rest before and after immersion into the water for normotensive, treated hypertensive and untreated hypertensive subjects.

Parameters	Normotensive		Treated hypertensive		Untreated hypertensive	
	Before	After	Before	After	Before	After
**HR (bpm)**	78±3	79±3	79±3	78±3	86±3	83±2
**SBP (mmHg)**	122±2	119±2	123±2	120±2	153±2*	150±2^‡^
**DBP (mmHg)**	81±2	80±2	80±2	80±2	91±1*	88±1^‡^
**MBP (mmHg)**	95±1	93±2	94±1	93±1	111±1	109±1
**HPP (bpm×mmHg)**	9497±387	9417±406	9659±301	9421±351	13074±459*	12497±387^‡^

Values are presented as mean *±* SD of normotensive, treated hypertensive and untreated hypertensive subjects for heart rate (HR), systolic blood pressure (SBP), diastolic blood pressure (DBP), mean blood pressure and heart rate pressure product (HPP). Two-way ANOVA was used, followed by Bonferroni’s post hoc test.

*p<0.001 indicates statistically significant differences between normotensive and treated hypertensive subjects on land.

^‡^p<0.001 indicates statistically significant differences between normotensive and treated hypertensive subjects in water.

**Fig 1:**
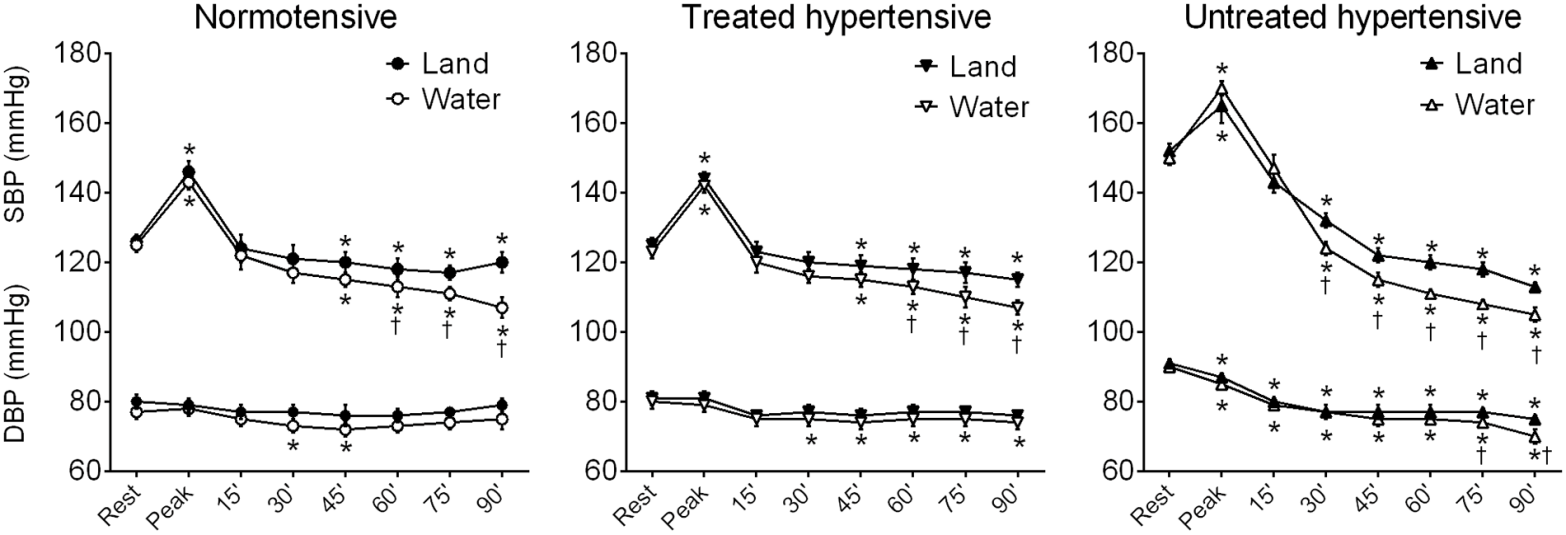
Effects of a single exercise session on the blood pressure. Systolic (SBP) and diastolic blood pressures (DBP) were evaluated at rest, at exercise peak and after 15, 30, 45, 60 and 90 minutes after land- or water-ergometry in groups Normotensive, Treated hypertensive and Untreated hypertensive. Values are presented in mean *±* SEM. *p<0.05 vs rest; ^†^p<0.05 vs. land-based protocol.

The analyses of land- and water-based exercise at rest, at exercise peak and during recovery (from 15 to 90 min) are presented in Fig 1. In both exercise modalities, a significant increase in SBP was observed from rest to peak exercise in all groups. In DBP, no changes occurred at the peak exercise in the N or TH groups, while a slight reduction was noted for the untreated hypertensive patients.

**Fig 2:**
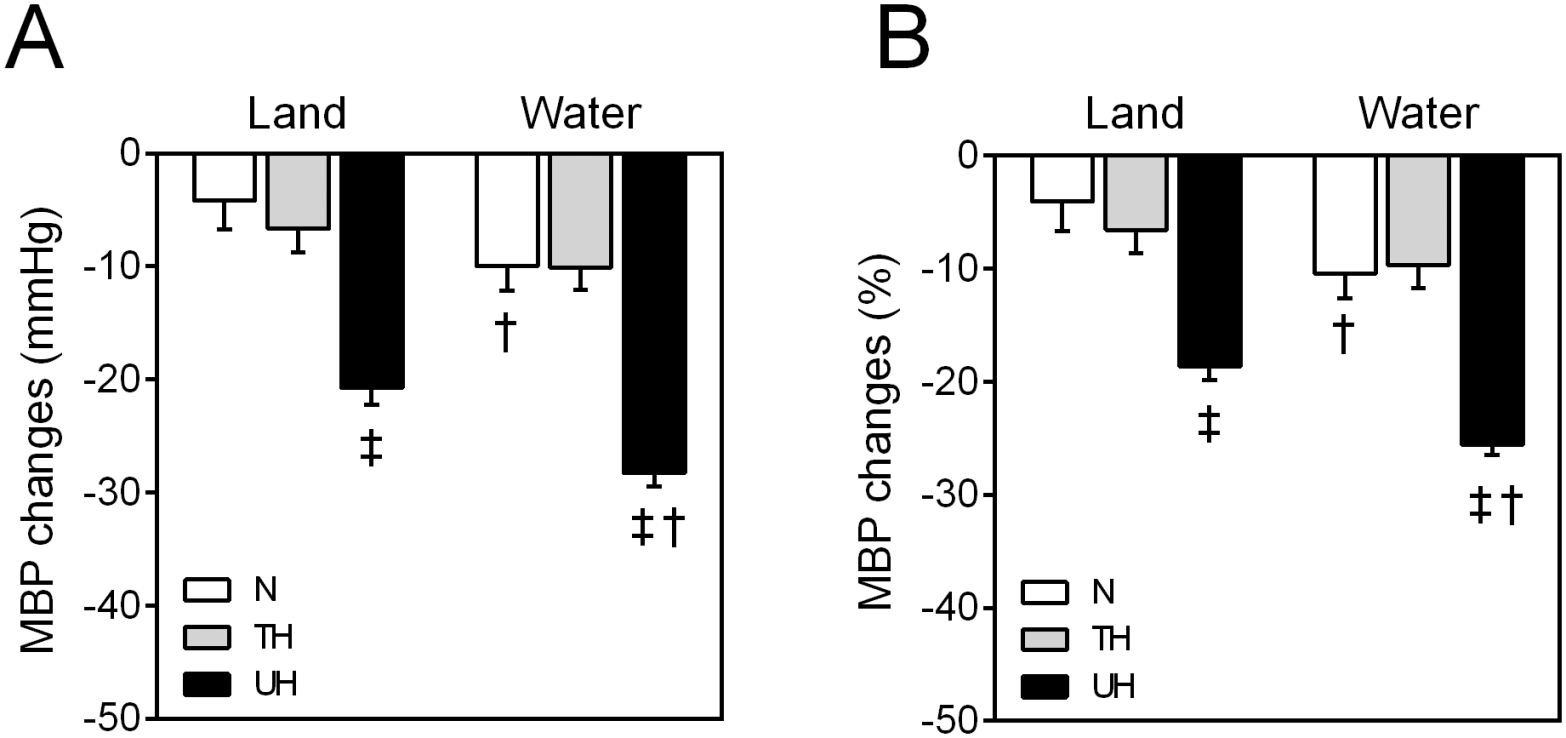
Absolute and percent of changes in the mean blood pressure after 90 minutes of the exercise session. A) Absolute of changes in the mean blood pressure (MBP) after exercise session land- or water-based in all groups. B) Percent of changes in the MBP. Values are presented in mean *±* SEM. ^‡^p<0.05 vs N and TH. ^†^p<0.05 vs land-based protocol. (Two-way ANOVA paired by protocol, followed by Bonferroni’s post-hoc test).

During recovery, the land-based protocol induced PEH, as indicated by decreasing SBP values after 45 min post-exercise in the N and TH groups and after 30 min post-exercise in the UH group compared to rest measures. However, in the water-based exercise protocol, PEH occurred as early as 30 min after the exercise session in all groups and reached higher hypotensive levels when compared to land-based PEH in the same recovery period. Ninety minutes after the exercise session, PEH was significantly higher in the water-based protocol; additionally, the BP was significantly decreased in relation to that measured at peak exercise. If compared to resting state, MBP decreased significantly more in UH patients than the other groups for both exercise modalities (Fig 2). Moreover, this hypotensive effect was greater when water-based exercise was performed in the N group (land: -4.1±2.5 mmHg (4% reduction) vs. water: -10.0±2.1 mmHg (10% reduction), p<0.05) and the UH group (land: -20.7±1.5 mmHg (18% reduction) vs. water: -28.3±1.1 mmHg (26% reduction), p<0.05).

Concerning DBP behavior after exercise protocols, while no statistical changes were noted in the land-based protocol in the N and TH groups when compared to rest, DBP was decreased in UH patients as early as 15 min after the exercise session. However, the water-based protocol led to a slight but significant reduction in the N group 30 and 45 min after the exercise session and in TH patients, from 30 to 90 min after the exercise session. Finally, although the water-based protocol induced a reduction in the DPB from 15 to 90 minutes after the exercise sessions, similar to a land-based program, exercise in the water led to a greater hypotensive effect 75 and 90 min post-exercise.

**Fig 3:**
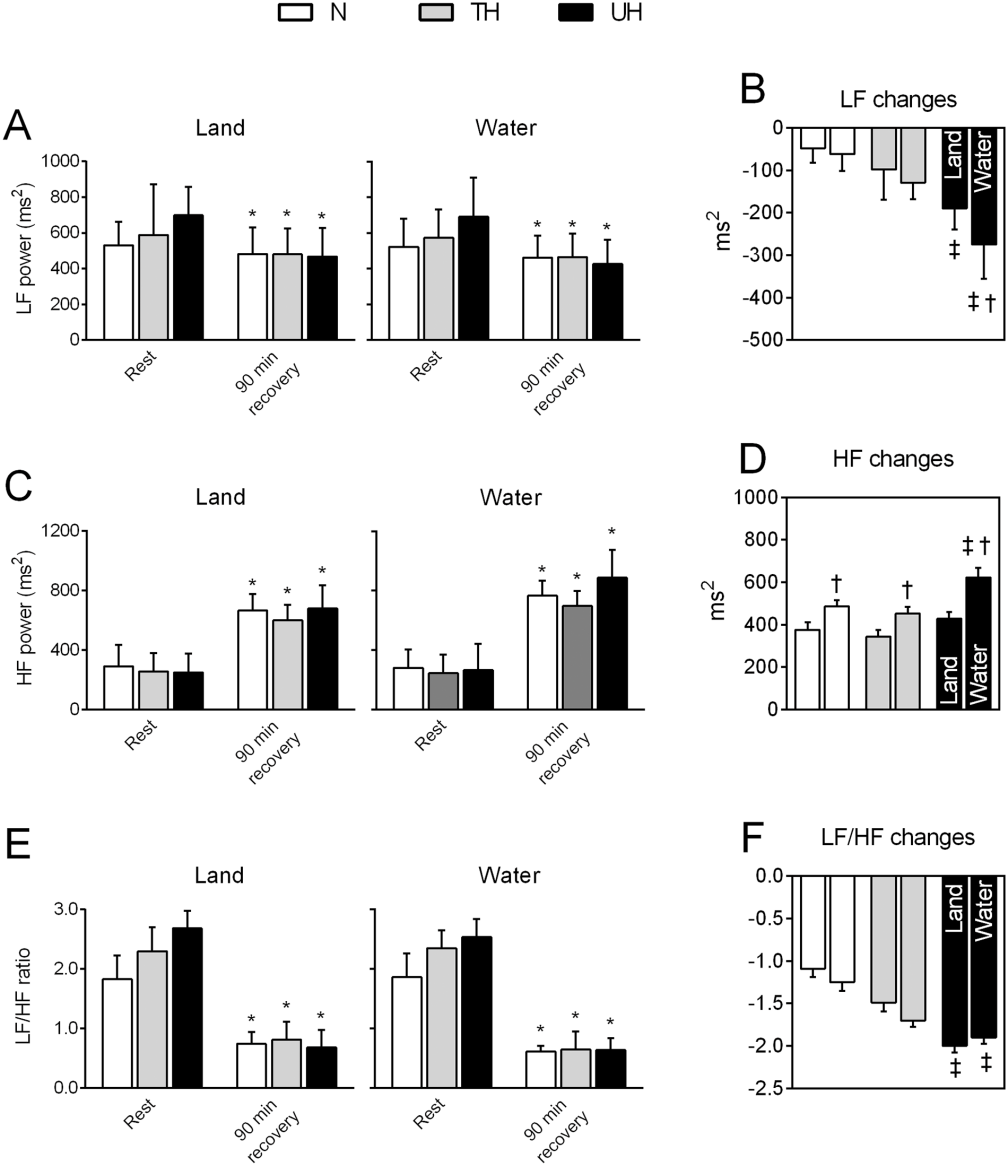
Heart rate variability changes at rest and after 90 minutes of recovery from land- and water-based exercise session. In A-B: the low frequency (LF) component, C-D: the high frequency (HF) component and, E-F: their ratio (LF/HF). N: normotensive individuals; TH: treated hypertensive patients; UH: untreated hypertensive patients. Values are mean ± SEM. *p<0.05 vs. rest (Two-way ANOVA paired by groups, followed by Bonferroni’s post-hoc test). ^‡^p<0.05 vs N and TH. ^†^p<0.05 vs land-based protocol. (Two-way ANOVA paired by protocol, followed by Bonferroni’s post-hoc test).

The changes in the HRV parameters assessed at rest and 90 min after exercise sessions are shown in Fig 3. At rest, the spectral bands suggested a predominant sympathetic tone in the UT, a trend to increased LF power (Fig 3A) and LF/HF ratio (Fig 3E) when compared with the normotensive individuals. After 90 minutes of exercise cessation, when compared to the respective baseline value, there was a significant decrease in the LF power and an increase in HF after both exercise modalities for all analyzed groups (Fig 3A and 3C). Moreover, the LF\HF ratio was significantly reduced 90 min after exercise sessions as well (Fig 3E). When comparing land-versus water-based efforts, the effects on the HRV parameters were greater if water cycling ergometry was performed; the effectiveness of this water-based exercise was significantly higher in the UH patients when compared to that in the TH and N subjects (Fig 3B, 3D and 3F).

## Discussion

This study analyzed the impact of land- and water-based exercise on hemodynamic parameters of normotensive and hypertensive subjects. Regular exercise practice is universally recommended to promote health as well as prevent several diseases. Furthermore, there is emerging evidence supporting the fact that a reduction in blood pressure levels resulting from aerobic exercise is primarily a result of the immediate response to a recent exercise session, i.e., PEH [20,32,33]. Among the elderly, the obese and those with low joint mobility [34], water-based exercise is increasingly popular and recommended. Furthermore, physical activities performed in the water have been prescribed for recovery and injury treatment in athletes [30] with significant cardiovascular benefits [19,20] and decreased exertion intensity [24,34].

To the best of our knowledge, this is the first study aimed to assess PEH in different clinical populations considering different environmental effects on physical exercise sessions. In the present study, higher blood pressure decay after both land- and water-based exercise was observed; however, special attention should address the water environment. The water-based exercise was able to promote an important decay in the MBP after 90 min of exercise by 10%, 10%, and 28% in normotensive, treated hypertensive and untreated hypertensive subjects, respectively. The PEH response of water-based exercise has been evaluated by few studies [15,16]. Rodriguez et al. [15] analyzed the PEH magnitude in normotensive and hypertensive women, describing higher effects after exercise was performed in water than on land; however, no mechanism was described. In contrast, Pontes Junior et al. [16] evaluated treated hypertensive subjects and found similar PHE responses after exercise on land and in water with no physiological marker differences for hypotensive effects between land and water sessions.

Additionally, our data demonstrated PEH after 45–60 minutes of land-based exercise, similar to that described in the literature [11], although slightly late when compared with the time that hypotension was observed. Notwithstanding, this effect of hypotensive response is closely related to the initial arterial blood pressure values [9], exercise intensity, modality and duration [9,10,11]. The effectiveness of PEH, even 24 hours after aerobic exercise, is well described with strong scientific evidence in both hypertensive [12,13,15] and normotensive [12] subjects. The main mechanism associated with PEH is the reduction of sympathetic activity [35], reflecting cardiac adrenergic receptor sensitivity attenuation, changes in renin and angiotensin release as a result of decreased catecholamine synthesis [36], lower stroke volume [37], peripheral vascular resistance [38] and synthesis of vasopressin [23] and endothelines [39].

In this study, the HRV of subjects from all groups was evaluated. The rhythmic activity of the sinoatrial node is mainly modulated by the autonomous nervous system, which exhibits a predominant vagal tone in most species, including humans. Since HR may significantly vary as a result of breathing, physical activity or mental stress, or as a reflex component in response to sudden changes in BP, it is well accepted that HRV quantifies the autonomic tone to the heart [40]. As a result, different parameters derived from the HRV analysis were modified in parallel with changes in sympathovagal balance [40].

Moreover, under pathophysiological conditions, a reduced HRV associated with a predominance of the sympathetic component occurs and has been identified as a strong indicator of high cardiovascular risk in healthy individuals and in patients with hypertension, myocardial infarction and atherosclerosis [40]. In our sample, the spectral bands resulting from HRV in the frequency domain assessed at rest suggested the predominance of sympathetic modulation in hypertension, as evidenced by increased LF power and consequent shifting toward a pathophysiological condition of sympathovagal balance (i.e., increased LF/HF) in untreated hypertensive patients compared to control subjects. In addition, it is worth noting that treated hypertensive patients exhibit a trend to changed HRV parameters without statistical significance, suggesting the close relationship between altered HRV and high blood pressure levels. These data reinforce that HRV is likely to be influenced by numerous factors, including the stage of hypertension and pharmacological treatment.

Although the literature is not completely consistent, evidence exists that aerobic exercise in healthy subjects and patients with disease can increase the HRV and vagal tone of the heart [41–43]. Considering a single session of exercise, as used in the present study, a significant drop in the LF component of power associated with an increased HF component can be observed in all groups after both exercise modalities. It is generally well accepted that while the HF band of the HFV spectrum is used to reflect the vagal activity, LF is attributed to modulation of both sympathetic and parasympathetic activities. Thus, although we have not assessed it at the peak of the exercise sessions, it was normally observed that overall, HRV parameters considerably decrease. However, during exercise recovery, HRV is progressively regained as a result of the sudden loss of the central command and baroreflex activation that contribute to this increased vagal tone and heart rate recovery observed throughout the entire recovery period. Additionally, withdrawal of the sympathetic tone and a progressive decrease in the blood pressure levels was noted, characterizing PEH [44].

Lovato et al. [45] reported that although LF decreases and HF increases during exercise recovery, differences have not been found for these HRV variables between rest and the 60-minute recovery period after a single session of aerobic plus resistance exercises in normotensive men. Conversely, it is important to note that the HF power we detected at 90 minutes of recovery after both land- and water-based ergometry was significantly greater than that assessed at rest, suggesting that more than just a parasympathetic reactivation in PEH occurs. Additionally, the increase in HF and the reduction in LF and LF/HF ratio during recovery were also significantly greater in the water- than in the land-based protocol, reinforcing the effectiveness of ergometry under immersion, especially for hypertensive patients. The exact underlying mechanisms by which exercise improves the vagal tone are not completely clarified. However, potential mediators are angiotensin II and nitric oxide (NO), both mechanisms enrolled in PEH. While angiotensin II, which is known to inhibit cardiac vagal tone, may be suppressed during exercise and after exercise, the increased NO bioavailability during exercise training could increase the vagal activity during recovery [43].

Among all cardiovascular variables, the behavior of blood pressure as measured in water-based exercise conditions is not well described, due to several technical difficulties, especially in procedures in which water is above the waist line. In fact, few studies showed conflicting results when the individual is immersed in the water for practicing exercise. Watenpaugh et al. [46] studied the cardiovascular responses in nine men and ten women for three hours; these subjects were sitting in water that was neck deep, with a temperature of 34 ± 0.1°C, showing that only the SBP increased approximately 10 to 12 mmHg in the second and third hour of immersion in both groups and reflecting an increase in pulse pressure. In a paper by Meyer and Bucking [47], no differences were found in BP under immersion in elderly patients with heart failure and control subjects. In contrast, Elvan-Taspinar et al. [48] observed decreased MAP and DAP in women undergoing immersion for three hours in a thermoneutral environment, suggesting that this response was associated with the decay of plasma renin activity and aldosterone response, leading to a decrease in peripheral vascular resistance.

Some data have shown that pulse pressure is directly influenced by immersion depth. Considering the immersion depth suggested by Graef and Kruel [49], protocols at the shoulders, chest xiphoid, umbilicus, hip and knee lines presented a reduction in the heart rate compared to the land condition. However, in the present study, no differences were found among levels of immersion; similar results were found to other publications [15,16].

In addition, other factors should be noted in water immersion exercise efforts, such as water temperature as described by Park et al. [50]. Significant changes in the hemodynamics were found in different water temperatures and immersion conditions, suggesting that increased venous return, central blood volume and cardiac output, associated with decreased peripheral resistance and changes in the autonomic responses may explain the influence of water temperature on the BP under immersion. Therefore, we aimed to control the water temperature to approximately 30±1°C in our study according to the AEA [31] recommendations for aquatic fitness practice and following published protocols [32].

In summary, our study demonstrated that water-ergometry exercise was effective in promoting a higher magnitude of PEH in older women compared to land-based exercise session, with more evident results obtained in hypertensive untreated patients. Although we believe that different intensities, durations and water immersion conditions and its long-term effects should be considered in further studies, the data presented here enlarge the discussion of distinct strategies for the prevention and treatment of hypertension. Specifically, water-based cycling is a potential non-pharmacological strategy to control blood pressure, considering its high effectiveness and low injury and cardiovascular risk. Despite the effectiveness of both protocols in promoting PEH, a special trend was observed in the UT group; the UT group exhibited a higher magnitude of PEH and HRV improvement in water- compared with land-based exercise. As a result, water based-exercise sessions may potentially be recommended for blood pressure control and autonomic modulation with a consequent reduction of cardiovascular risk, especially due to the PEH-related effects.

## Supporting information

S1 Fig Graphical abstract for educational purposes. (PDF)

S1 File Data on changes in blood pressure during and after the exercise session. (XLSX)

S2 File Individual data of absolute and percent changes in blood pressure. (XLSX)

S3 File Data on heart rate variability in all groups. (XLSX)
