# Supplementary material for: Post-exercise hypotension and heart rate variability response after water- and land-ergometry exercise in hypertensive patients
Source: PLoS One. 2017 Jun 28;12(6):e0180216. doi: 10.1371/journal.pone.0180216 (PMC5489191; doi:10.1371/journal.pone.0180216)
Supplement: S1 Fig — (PDF) [file pone.0180216.s001.pdf]

Post-exercise hypotension in Hypertensive patients

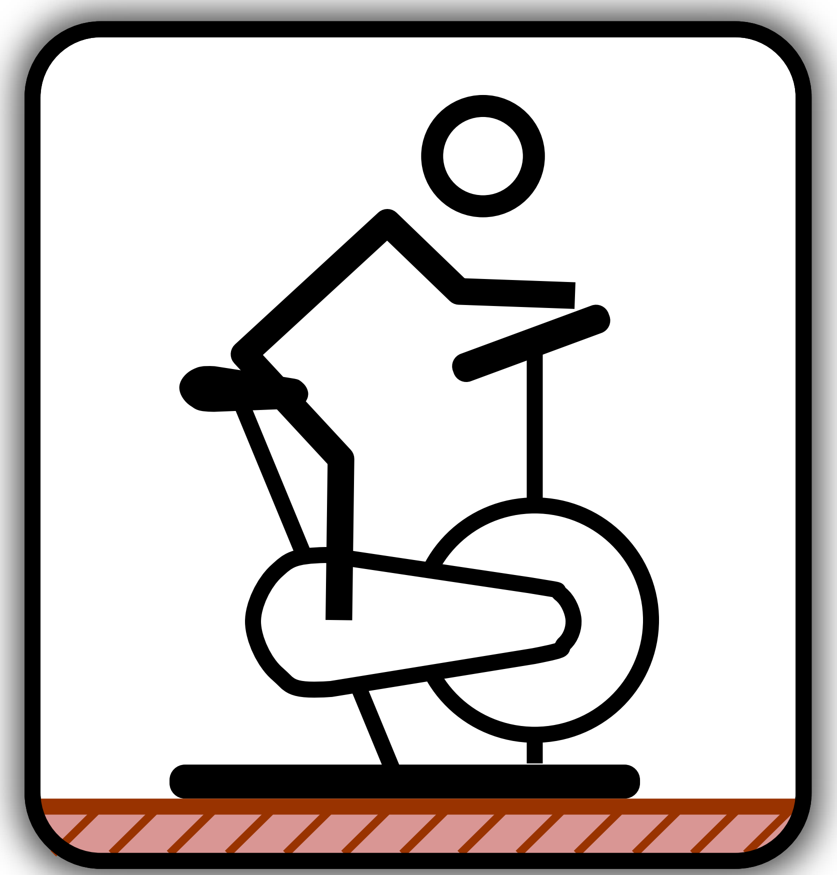

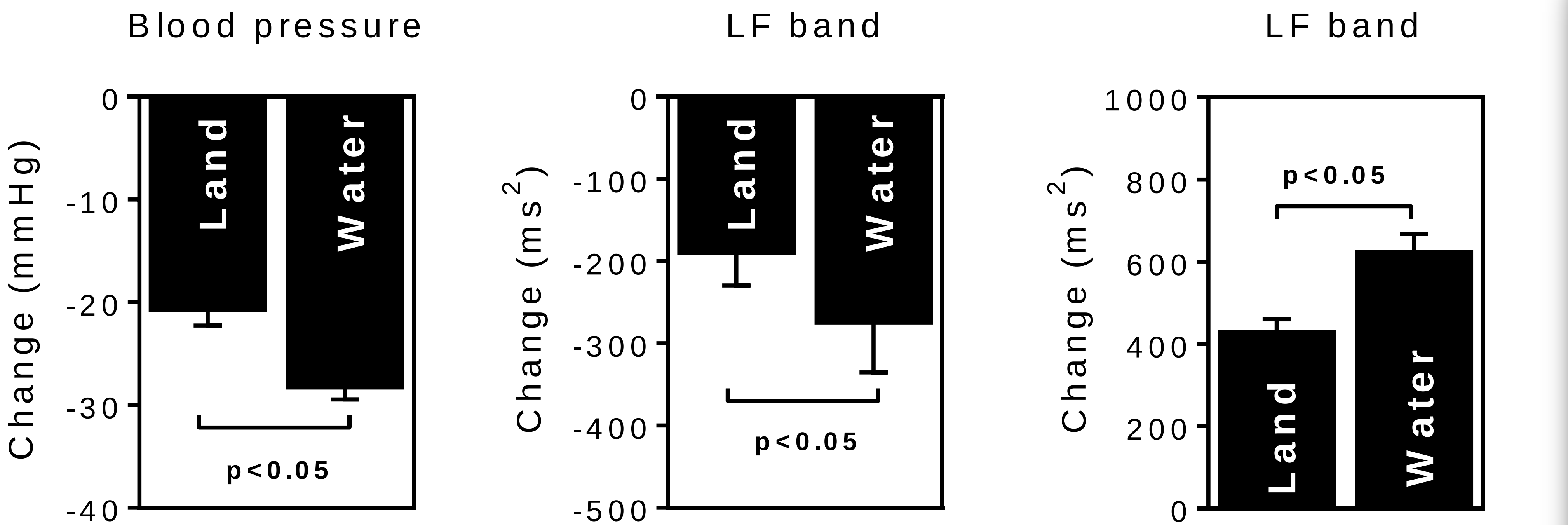

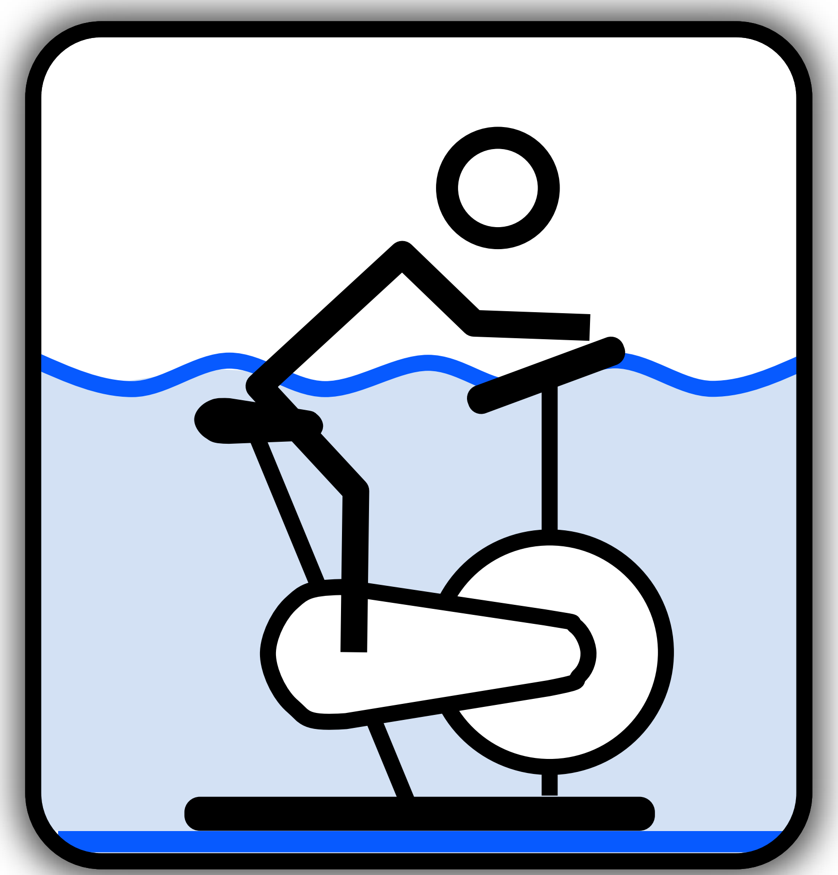

Land-based *versus* Water-based

Ergometric cycling
